# Supplementary material for: Personalizing the decision of dabigatran versus warfarin in atrial fibrillation: A secondary analysis of the Randomized Evaluation of Long-term anticoagulation therapY (RE-LY) trial
Source: PLoS One. 2021 Aug 19;16(8):e0256338. doi: 10.1371/journal.pone.0256338 (PMC8376053; doi:10.1371/journal.pone.0256338)
Supplement: S1 Fig — (DOCX) [file pone.0256338.s001.docx]

**S1 Fig. Calibration curve for model predicting stroke or systemic embolism.**

Prediction Model Calibration for Stroke/Systemic Embolism
