# Supplementary material for: Personalizing the decision of dabigatran versus warfarin in atrial fibrillation: A secondary analysis of the Randomized Evaluation of Long-term anticoagulation therapY (RE-LY) trial
Source: PLoS One. 2021 Aug 19;16(8):e0256338. doi: 10.1371/journal.pone.0256338 (PMC8376053; doi:10.1371/journal.pone.0256338)
Supplement: S1 Table — (DOCX) [file pone.0256338.s003.docx]

**S1 Table. Baseline Characteristics of RE-LY Patients by Treatment Group**

|  | **Treatment Group** | | | **Total** |
| --- | --- | --- | --- | --- |
|  | **Dabigatran, 110mg n = 5983** | **Dabigatran, 150mg n = 6059** | **Warfarin n = 5998** | **n = 18040** |
| **Age, years** |  |  |  |  |
| Mean ± SD* | 71.3 ± 8.6 | 71.4 ± 8.7 | 71.5 ± 8.5 | 71.4 ± 8.6 |
| Median (IQR†) | 72.0 (67.0, 77.0) | 72.0 (67.0, 78.0) | 72.0 (67.0, 77.0) | 72.0 (67.0, 77.0) |
| **Age Categories, years** |  |  |  |  |
| <40 | 21 (0.4%) | 26 (0.4%) | 12 (0.2%) | 59 (0.3%) |
| ≥40 and <50 | 99 (1.7%) | 86 (1.4%) | 89 (1.5%) | 274 (1.5%) |
| ≥50 and <65 | 873 (14.6%) | 916 (15.1%) | 849 (14.2%) | 2638 (14.6%) |
| ≥65 and <75 | 2655 (44.4%) | 2574 (42.5%) | 2635 (43.9%) | 7864 (43.6%) |
| ≥75 | 2335 (39.0%) | 2457 (40.6%) | 2413 (40.2%) | 7205 (39.9%) |
| **Sex** |  |  |  |  |
| Female | 2130 (35.6%) | 2228 (36.8%) | 2202 (36.7%) | 6560 (36.4%) |
| **Ethnicity** |  |  |  |  |
| White | 4191 (70.0%) | 4258 (70.3%) | 4181 (69.7%) | 12630 (70.0%) |
| Asian | 948 (15.8%) | 961 (15.9%) | 955 (15.9%) | 2864 (15.9%) |
| Black | 51 (0.9%) | 57 (0.9%) | 66 (1.1%) | 174 (1.0%) |
| Other | 793 (13.3%) | 783 (12.9%) | 796 (13.3%) | 2372 (13.1%) |
| **Region** |  |  |  |  |
| United States, Canada | 2150 (35.9%) | 2195 (36.2%) | 2152 (35.9%) | 6497 (36.0%) |
| Western Europe | 1541 (25.8%) | 1552 (25.6%) | 1543 (25.7%) | 4636 (25.7%) |
| Asia | 918 (15.3%) | 929 (15.3%) | 926 (15.4%) | 2773 (15.4%) |
| Central Europe | 703 (11.7%) | 704 (11.6%) | 706 (11.8%) | 2113 (11.7%) |
| Latin America | 319 (5.3%) | 319 (5.3%) | 316 (5.3%) | 954 (5.3%) |
| Other | 352 (5.9%) | 360 (5.9%) | 355 (5.9%) | 1067 (5.9%) |
| **Atrial Fibrillation Type** |  |  |  |  |
| Paroxysmal | 1916 (32.0%) | 1974 (32.6%) | 2030 (33.9%) | 5920 (32.8%) |
| Persistent | 1941 (32.5%) | 1901 (31.4%) | 1922 (32.0%) | 5764 (32.0%) |
| Permanent | 2123 (35.5%) | 2183 (36.0%) | 2045 (34.1%) | 6351 (35.2%) |
| **Aspirin use at baseline** |  |  |  |  |
|  | 2384 (39.8%) | 2338 (38.6%) | 2431 (40.5%) | 7153 (39.7%) |
| **CHADS2‡ Score** |  |  |  |  |
| Mean ± SD | 2.1 ± 1.1 | 2.2 ± 1.1 | 2.1 ± 1.1 | 2.1 ± 1.1 |
| Median (IQR) | 2.0 (1.0, 3.0) | 2.0 (1.0, 3.0) | 2.0 (1.0, 3.0) | 2.0 (1.0, 3.0) |
| **CHADS2 Score** |  |  |  |  |
| 0 | 151 (2.5%) | 145 (2.4%) | 155 (2.6%) | 451 (2.5%) |
| 1 | 1797 (30.0%) | 1810 (29.9%) | 1705 (28.4%) | 5312 (29.4%) |
| 2 | 2081 (34.8%) | 2129 (35.1%) | 2212 (36.9%) | 6422 (35.6%) |
| 3+ | 1954 (32.7%) | 1975 (32.6%) | 1926 (32.1%) | 5855 (32.5%) |
| **History of Heart Failure** |  |  |  |  |
|  | 1929 (32.2%) | 1930 (31.9%) | 1915 (31.9%) | 5774 (32.0%) |
| **Baseline Heart Failure Classification** |  |  |  |  |
| NYHA**§** I | 293 (15.2%) | 292 (15.1%) | 295 (15.4%) | 880 (15.3%) |
| NYHA II | 1222 (63.4%) | 1195 (62.0%) | 1219 (63.7%) | 3636 (63.0%) |
| NYHA III | 383 (19.9%) | 400 (20.7%) | 352 (18.4%) | 1135 (19.7%) |
| NYHA IV | 30 (1.6%) | 41 (2.1%) | 48 (2.5%) | 119 (2.1%) |
| **LVEF**\|\| |  |  |  |  |
| <=40% | 647 (22.0%) | 651 (21.9%) | 628 (21.2%) | 1926 (21.7%) |
| **Baseline Hypertension**  **Requiring Medical Treatment** |  |  |  |  |
|  | 4711 (78.7%) | 4781 (78.9%) | 4729 (78.8%) | 14221 (78.8%) |
| **History of Diabetes Mellitus** |  |  |  |  |
|  | 1401 (23.4%) | 1398 (23.1%) | 1405 (23.4%) | 4204 (23.3%) |
| **History of Stroke/Systemic Embolism/TIA#** |  |  |  |  |
|  | 1302 (21.8%) | 1357 (22.4%) | 1282 (21.4%) | 3941 (21.8%) |
| **Baseline Creatinine Clearance [mL/min]** |  |  |  |  |
| Mean ± SD | 73.0 ± 27.7 | 72.8 ± 28.2 | 73.0 ± 27.4 | 72.9 ± 27.8 |
| Median (IQR) | 68.7 (53.2, 87.2) | 67.9 (53.0, 86.4) | 68.5 (53.8, 86.6) | 68.4 (53.4, 86.8) |
| **Creatinine Clearance Grouping** |  |  |  |  |
| <30 | 14 (0.2%) | 31 (0.5%) | 29 (0.5%) | 74 (0.4%) |
| ≥30 and <50 | 1127 (19.7%) | 1152 (19.7%) | 1048 (18.2%) | 3327 (19.2%) |
| ≥50 and <80 | 2705 (47.2%) | 2770 (47.5%) | 2794 (48.7%) | 8269 (47.8%) |
| ≥80 | 1889 (32.9%) | 1880 (32.2%) | 1872 (32.6%) | 5641 (32.6%) |
| **Weight, kg** |  |  |  |  |
| Mean ± SD | 82.9 ± 19.9 | 82.4 ± 19.3 | 82.6 ± 19.6 | 82.7 ± 19.6 |
| Median (IQR) | 80.5 (70.0, 94.0) | 80.0 (69.0, 93.0) | 80.0 (70.0, 93.0) | 80.0 (69.9, 93.4) |
|  |  |  |  |  |

**^*^**SD, standard deviation

†IQR, interquartile range

**‡** CHADS2, Congestive Heart Failure, Hypertension, Age, Diabetes, Stroke (2)

**§**NYHA, New York Heart Association

||LVEF, left ventricular ejection fraction

#TIA, transient ischemic attack
