# Supplementary material for: Personalizing the decision of dabigatran versus warfarin in atrial fibrillation: A secondary analysis of the Randomized Evaluation of Long-term anticoagulation therapY (RE-LY) trial
Source: PLoS One. 2021 Aug 19;16(8):e0256338. doi: 10.1371/journal.pone.0256338 (PMC8376053; doi:10.1371/journal.pone.0256338)
Supplement: S2 Table — (DOCX) [file pone.0256338.s004.docx]

**S2 Table.** Primary Outcomes of Stroke or Systemic Embolism and Major Bleeding by Treatment Group.

|  | **Treatment Pattern Label** | | | **Total** | **P-Value** |
| --- | --- | --- | --- | --- | --- |
|  | **Dabigatran 110mg n = 5983** | **Dabigatran 150mg n = 6059** | **Warfarin n = 5998** | **n = 18040** |  |
| **Stroke/Systemic Embolism** |  |  |  |  | < 0.001 |
|  | 84 (1.4%) | 53 (0.9%) | 125 (2.1%) | 262 (1.5%) |  |
| **Major Bleeding** |  |  |  |  | 0.011 |
|  | 216 (3.6%) | 261 (4.3%) | 281 (4.7%) | 758 (4.2%) |  |
