# Supplementary material for: Personalizing the decision of dabigatran versus warfarin in atrial fibrillation: A secondary analysis of the Randomized Evaluation of Long-term anticoagulation therapY (RE-LY) trial
Source: PLoS One. 2021 Aug 19;16(8):e0256338. doi: 10.1371/journal.pone.0256338 (PMC8376053; doi:10.1371/journal.pone.0256338)
Supplement: S3 Table — (DOCX) [file pone.0256338.s005.docx]

**S3 Table. Integer Score for Stroke or Systemic Embolism Risk.** Each treatment category has a number of starting points, to which the integer values below are added based on the patient’s clinical and demographic traits. Higher integer scores indicate higher risk of stroke or systemic embolism.

|  | **Treatment Group** | | |
| --- | --- | --- | --- |
|  | **Dabigatran, 110mg** | **Dabigatran, 150mg** | **Warfarin** |
| **Starting Points** | 6 | -59 | 0 |
| **Age Categories, years** |  |  |  |
| <50 | 0 | 0 | 0 |
| 50-59 | -2 | 5 | 0 |
| 60-69 | -3 | 10 | 0 |
| 70-79 | -5 | 15 | 0 |
| ≥80 | -6 | 19 | 1 |
| **Diabetes** | 1 | 1 | 7 |
| **Prior Stroke** | 1 | 1 | 7 |
| **Creatinine Clearance, ml/min** |  |  |  |
| <50 | 0 | 0 | 0 |
| 50-79 | -2 | 2 | -3 |
| ≥80 | -4 | 3 | -6 |
| **Region** |  |  |  |
| United States, Canada | 0 | 0 | 0 |
| Western Europe | 1 | 1 | 1 |
| Asia | 4 | 4 | 4 |
| Central Europe | 0 | 0 | 0 |
| Latin America | 1 | 1 | 1 |
| Other | 1 | 1 | 1 |
