# Supplementary material for: Personalizing the decision of dabigatran versus warfarin in atrial fibrillation: A secondary analysis of the Randomized Evaluation of Long-term anticoagulation therapY (RE-LY) trial
Source: PLoS One. 2021 Aug 19;16(8):e0256338. doi: 10.1371/journal.pone.0256338 (PMC8376053; doi:10.1371/journal.pone.0256338)
Supplement: S4 Table — (DOCX) [file pone.0256338.s006.docx]

**S4 Table. Integer Score for Major Bleeding Risk.** Each treatment category has a number of starting points, to which the integer values below are added based on the patient’s clinical and demographic traits. Higher integer scores indicate higher risk of major bleeding.

|  | **Treatment Group** | | |
| --- | --- | --- | --- |
|  | **Dabigatran, 110mg** | **Dabigatran, 150mg** | **Warfarin** |
| **Starting Points** | -38 | -60 | 0 |
|  |  |  |  |
| **Age Categories, years** |  |  |  |
| <50 | 0 | 0 | 0 |
| 50-59 | 5 | 7 | 1 |
| 60-69 | 9 | 13 | 2 |
| 70-79 | 14 | 20 | 3 |
| ≥80 | 18 | 26 | 4 |
| **Diabetes** | 3 | 3 | 3 |
| **Prior Stroke** | 2 | 2 | 2 |
| **Heart Failure** | 4 | 0 | 1 |
| **Aspirin Use** | 2 | 2 | 2 |
| **Creatinine Clearance, ml/min** |  |  |  |
| <50 | 0 | 0 | 0 |
| 50-79 | -3 | -3 | -3 |
| ≥80 | -6 | -6 | -6 |
| **Region** |  |  |  |
| United States, Canada | 0 | 0 | 0 |
| Western Europe | -6 | -6 | -6 |
| Asia | -5 | -5 | -5 |
| Central Europe | -8 | -8 | -8 |
| Latin America | -6 | -6 | -6 |
| Other | -6 | -6 | -6 |
| **Atrial Fibrillation Type** |  |  |  |
| Paroxysmal | 2 | 2 | -2 |
| Persistent | 0 | 0 | 0 |
| Permanent | 1 | 2 | 1 |
| **Weight, pounds** |  |  |  |
| <120 | 0 | 0 | 0 |
| 120-159 | 5 | 8 | 2 |
| 160-199 | 10 | 17 | 4 |
| 200-249 | 16 | 27 | 6 |
| ≥250 | 22 | 39 | 8 |
