# Supplementary material for: Personalizing the decision of dabigatran versus warfarin in atrial fibrillation: A secondary analysis of the Randomized Evaluation of Long-term anticoagulation therapY (RE-LY) trial
Source: PLoS One. 2021 Aug 19;16(8):e0256338. doi: 10.1371/journal.pone.0256338 (PMC8376053; doi:10.1371/journal.pone.0256338)
Supplement: S1 Appendix — (DOCX) [file pone.0256338.s007.docx]

**S1 Appendix.** **Definition of increased risk of stroke.**

Any one of the following risk factors:

a. History of previous stroke, transient ischemic attack, or systemic embolism

b. Ejection fraction less than 40% documented by echocardiogram, radionuclide or contrast angiogram in the last 6 months

c. Symptomatic heart failure, New York Heart Association class 2 or higher in the last 6 months

d. Age at least 75 years

e. Age at least 65 years and one of the following:

i. Diabetes mellitus on treatment

ii. Documented coronary artery disease (any of: prior myocardial infarction, positive stress test, positive nuclear perfusion study, prior CABG surgery or PCI, or angiogram showing at least 75% stenosis in a major coronary artery)

iii. Hypertension requiring medical treatment
