# Supplementary material for: Personalizing the decision of dabigatran versus warfarin in atrial fibrillation: A secondary analysis of the Randomized Evaluation of Long-term anticoagulation therapY (RE-LY) trial
Source: PLoS One. 2021 Aug 19;16(8):e0256338. doi: 10.1371/journal.pone.0256338 (PMC8376053; doi:10.1371/journal.pone.0256338)
Supplement: S2 Appendix — (DOCX) [file pone.0256338.s008.docx]

**S2 Appendix. Definition of increased risk for hemorrhage.**

Conditions associated with an increased risk of bleeding:

a. Major surgery within the previous month

b. Planned surgery or intervention within the next 3 months

c. History of intracranial, intraocular, spinal, retroperitoneal or atraumatic intra‐articular

bleeding

d. Gastrointestinal hemorrhage within the past year

e. Symptomatic or endoscopically documented gastroduodenal ulcer disease in the

previous 30 days

f. Hemorrhagic disorder or bleeding diathesis

g. Need for anticoagulant treatment of disorders other than atrial fibrillation

h. Fibrinolytic agents within 48 hours of study entry

i. Uncontrolled hypertension (systolic blood pressure greater than 180 mm Hg and/or

diastolic blood pressure greater than 100 mm Hg)

j. Recent malignancy or radiation therapy (within 6 months) and not expected to survive 3

years
