# Supplementary material for: Personalizing the decision of dabigatran versus warfarin in atrial fibrillation: A secondary analysis of the Randomized Evaluation of Long-term anticoagulation therapY (RE-LY) trial
Source: PLoS One. 2021 Aug 19;16(8):e0256338. doi: 10.1371/journal.pone.0256338 (PMC8376053; doi:10.1371/journal.pone.0256338)
Supplement: S3 Appendix — (DOCX) [file pone.0256338.s009.docx]

**S3 Appendix.** **Definition of primary endpoints.**

Stroke was defined as the sudden onset of a focal neurologic deficit in a location consistent

with the territory of a major cerebral artery and categorized as ischemic, hemorrhagic, or

unspecified.

Hemorrhagic transformation of ischemic stroke was not considered to be hemorrhagic

stroke. Intracranial hemorrhage consisted of hemorrhagic stroke and subdural or subarachnoid

hemorrhage.

Systemic embolism was defined as an acute vascular occlusion of an extremity or organ, documented by means of imaging, surgery, or autopsy.

Major bleeding was defined as a reduction in the hemoglobin level of at least 20 g per liter, transfusion of at least 2 units of blood, or symptomatic bleeding in a critical area or organ.
