# Supplementary material for: Personalizing the decision of dabigatran versus warfarin in atrial fibrillation: A secondary analysis of the Randomized Evaluation of Long-term anticoagulation therapY (RE-LY) trial
Source: PLoS One. 2021 Aug 19;16(8):e0256338. doi: 10.1371/journal.pone.0256338 (PMC8376053; doi:10.1371/journal.pone.0256338)
Supplement: S4 Appendix — (DOCX) [file pone.0256338.s010.docx]

**S4 Appendix.** **Variables included in stroke/systemic embolism risk model.**

a. Initial variables (15): treatment pattern, age, weight, sex, region, aspirin use at baseline, atrial fibrillation type, heart failure, hypertension, diabetes mellitus, prior stroke/embolus/transient ischemic attack, creatinine clearance, age interaction with treatment pattern, diabetes mellitus interaction with treatment pattern, creatinine clearance interaction with treatment pattern

b. final variables (9): treatment pattern, age, region, diabetes mellitus, prior stroke/embolus/transient ischemic attack, creatinine clearance, age interaction with treatment pattern, diabetes mellitus interaction with treatment pattern, creatinine clearance interaction with treatment pattern
