# Supplementary material for: Personalizing the decision of dabigatran versus warfarin in atrial fibrillation: A secondary analysis of the Randomized Evaluation of Long-term anticoagulation therapY (RE-LY) trial
Source: PLoS One. 2021 Aug 19;16(8):e0256338. doi: 10.1371/journal.pone.0256338 (PMC8376053; doi:10.1371/journal.pone.0256338)
Supplement: S5 Appendix — (DOCX) [file pone.0256338.s011.docx]

**S5 Appendix. Variables included in major bleeding risk model.**

a. Initial variables (16): treatment pattern, age, weight, sex, region, aspirin use at baseline, atrial fibrillation type, heart failure, hypertension, diabetes mellitus, prior stroke/embolus/transient ischemic attack, creatinine clearance, age interaction with treatment pattern, weight interaction with treatment pattern, atrial fibrillation type interaction with treatment pattern, heart failure interaction with treatment pattern

b. final variables (14): treatment pattern, age, weight, region, aspirin use at baseline, atrial fibrillation type, heart failure, diabetes mellitus, prior stroke/embolus/transient ischemic attack, creatinine clearance, age interaction with treatment pattern, weight interaction with treatment pattern, atrial fibrillation type interaction with treatment pattern, heart failure interaction with treatment pattern
